# Light‐Induced Uncaging for Time‐Resolved Observations of Biochemical Reactions by MAS NMR Spectroscopy

**DOI:** 10.1002/chem.202000770

**Published:** 2020-05-13

**Authors:** Julian de Mos, Andreas Jakob, Johanna Becker‐Baldus, Alexander Heckel, Clemens Glaubitz

**Affiliations:** ^1^ Institute for Biophysical Chemistry and Centre for Biomolecular Magnetic Resonance Goethe University Frankfurt Max-von-Laue-Str. 9 60438 Frankfurt am Main Germany; ^2^ Institute for Organic Chemistry and Chemical Biology Goethe University Frankfurt Max-von-Laue-Str. 7 60438 Frankfurt am Main Germany

**Keywords:** caged ATP, caged diacylglycerol, DgkA, enzymes, NMR spectroscopy, solid-state NMR

## Abstract

Light‐induced activation of biomolecules by uncaging of photolabile protection groups has found many applications for triggering biochemical reactions with minimal perturbations directly within cells. Such an approach might also offer unique advantages for solid‐state NMR experiments on membrane proteins for initiating reactions within or at the membrane directly within the closed MAS rotor. Herein, we demonstrate that the integral membrane protein *E. coli* diacylglycerol kinase (DgkA), which catalyzes the phosphorylation of diacylglycerol, can be controlled by light under MAS‐NMR conditions. Uncaging of NPE‐ATP or of lipid substrate NPE‐DOG by in situ illumination triggers its enzymatic activity, which can be monitored by real‐time ^31^P‐MAS NMR. This proof‐of‐concept illustrates that combining MAS‐NMR with uncaging strategies and illumination methods offers new possibilities for controlling biochemical reactions at or within lipid bilayers.

## Graphical abstract

**Engineering light control**: Light‐induced release of caged substrates to initiate enzymatic activity of the membrane enzyme diacylglycerol kinase. A first demonstration of in situ light control for biomolecular solid‐state NMR, highlighting its potential advantages to initiate reactions in membrane systems (see figure).

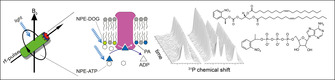

The development of light‐induced uncaging strategies for biochemical substrates, which have been inactivated with a photolabile protection group, enables a range of experiments with high spatial and temporal resolution especially in the cellular context. A broad set of tools for the caging of biologically relevant compounds has been developed enabling to address a wide range of biological applications.^1^ The high versatility of the uncaging approach with respect to temporal control and wavelength selectivity also provides a variety of opportunities for NMR spectroscopy or other biophysical methods for in situ triggering of enzymatic reactions, folding events or oligomerization/complex formation. In a molecular biophysical context, caged compounds have been utilized for example in NMR spectroscopy of soluble samples to induce folding of proteins, DNA and RNA or for enzyme studies.^2^ Also lipids have been an important target for developing uncaging strategies.1f, 3 Solid‐state NMR is extensively used for the investigation of lipids and membrane proteins within intact bilayers, but uncaging has not been explored yet for these applications. It could be advantageous because solid‐state NMR relies on fast sample rotation at the magic angle using sealed rotors, which makes the addition of substrates during the experiment almost impossible. Pre‐mixing before the NMR experiment followed by fast sample transfer into the magnet is in principle possible but requires tailoring of the experimental conditions towards slow kinetics and relies on a good distribution of the substrate within a heterogeneous proteoliposome sample. This becomes especially challenging for example for hydrophobic compounds such as lipid substrates or for targeting binding sites within the lumen of sealed liposomes.

To test whether these limitations could indeed be addressed by uncaging, we have chosen the *E. coli* membrane protein diacylglycerol kinase (DgkA), which phosphorylates diacylglycerol under ATP consumption (Figure 1). Its homotrimeric structure was determined by X‐ray crystallography in lipidic cubic phases.^4^ Its interfacial enzymatic reaction has been observed with time‐resolved ^31^P‐MAS NMR^5^ and its secondary structure,^6^ trimer symmetry and protomer interactions within the lipid bilayer were resolved by 3D‐ and DNP‐enhanced MAS‐NMR.^7^

**Figure 1:**
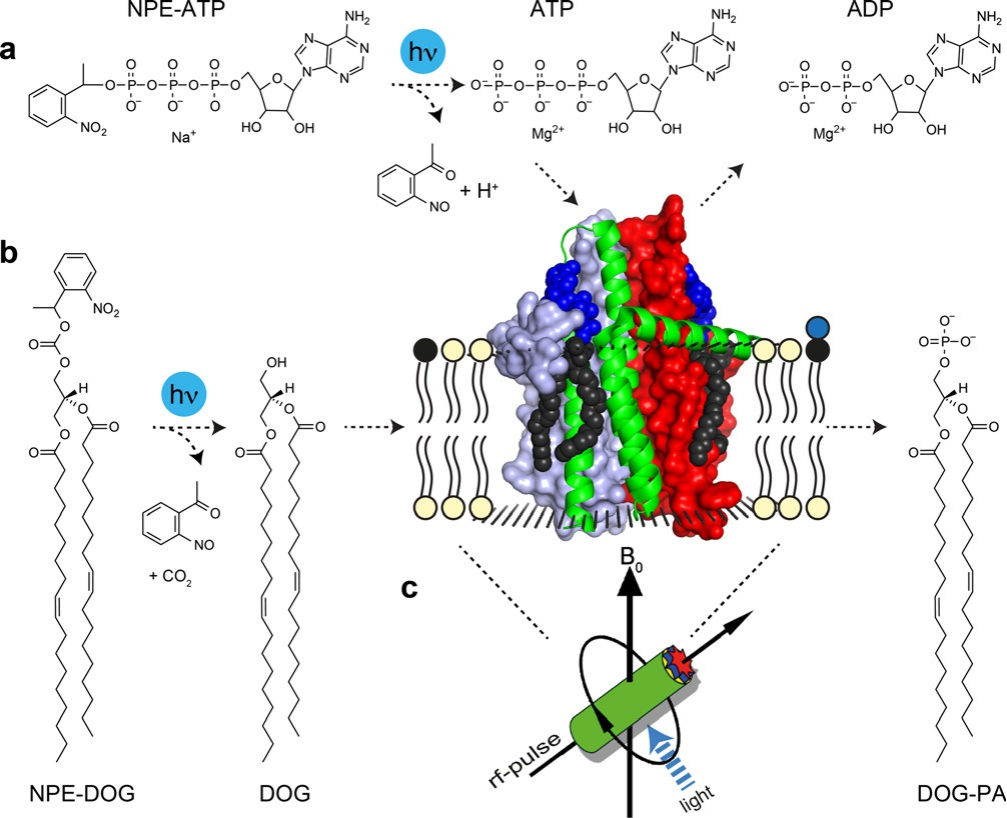
Light‐dependent approaches to initiate the enzymatic activity of DgkA in liposomes in situ under MAS‐NMR conditions by either a) uncaging the required nucleotide NPE‐ATP or by b) releasing the lipid substrate NPE‐dioleoylglycerol (NPE‐DOG). c) Uncaging requires efficient in situ sample illumination under MAS‐NMR conditions (see Figure S1 for further details). The DgkA structure cartoon was created from PDB 3ZE4.^4^

In mammalian cells, DAGs act as second messenger and get phosphorylated by lipid kinases, which are structurally rather distinct from the *E. coli* variant. Altered functions of individual DgkA isoforms have been implicated in a range of diseases, which requires a better understanding of their function.^8^ Therefore, developing tools by which such reactions could be studied directly within the membrane interface could have wide implications.

Here, the DgkA activity has been controlled by either uncaging NPE‐ATP (Figure 1 a) or by releasing NPE‐DOG, a DAG variant (Figure 1 b). Therefore, a robust and cost‐effective illumination setup for efficient in situ illumination under MAS at a high magnetic field was established. Similar to the illumination setup previously described for photo‐CIDNP,^9^ a fiber bundle with a macor ferrule was inserted into the MAS stator from beneath through a hole in the coil pedestal (Figure 1 c). A stretched radiofrequency coil geometry was used to enable efficient illumination of the sample volume, which was restricted to 15 μL in the center of the MAS rotor by insertion of rubber disks (Figure S1).

Using a high radiance UV LED as light source with a peak wavelength of 365 nm, 20 mW of UV light were available at the end of the fiber bundle for sample illumination. This setup was first tested on a sample containing DOPC liposomes and NPE‐ATP. Within minutes, NPE‐ATP could be successfully uncaged under MAS‐NMR conditions and the reaction could be monitored by ^31^P‐NMR direct detection (Figure S2). As the available signal‐to‐noise ratio limits the time resolution, the observed uncaging rate should be sufficient for a range of applications. However, liposomes as well as the uncaged NPE‐group absorb light and therefore reduce the uncaging efficiency at these concentrations to approx. 80 %.

Uncaging of NPE‐ATP was then carried out in the presence of DgkA (Figure 2) to induce its enzymatic activity. The lipid substrate DAG or other long‐chain variants were omitted initially, so that only basal ATP hydrolysis occurs.^5^, ^10^ Indeed, as shown in Figure 2 a, uncaging of NPE‐ATP by light is followed by ATP consumption and build‐up of ADP and Pi. Build‐up rates are comparable to those observed by us before under similar conditions but without uncaging of NPE‐ATP.^5^ Within 5 min an uncaging efficiency of approx. 65 % was achieved (see Figure S2). The amount of DgkA within the liposomes was chosen so that the bulk turnover of uncaged ATP by DgkA is significantly slower than the uncaging reaction. As a control, the same experiment was performed in the absence of Mg^2+^ for which uncaging but no ATP hydrolysis could be observed (Figure S3).

**Figure 2:**
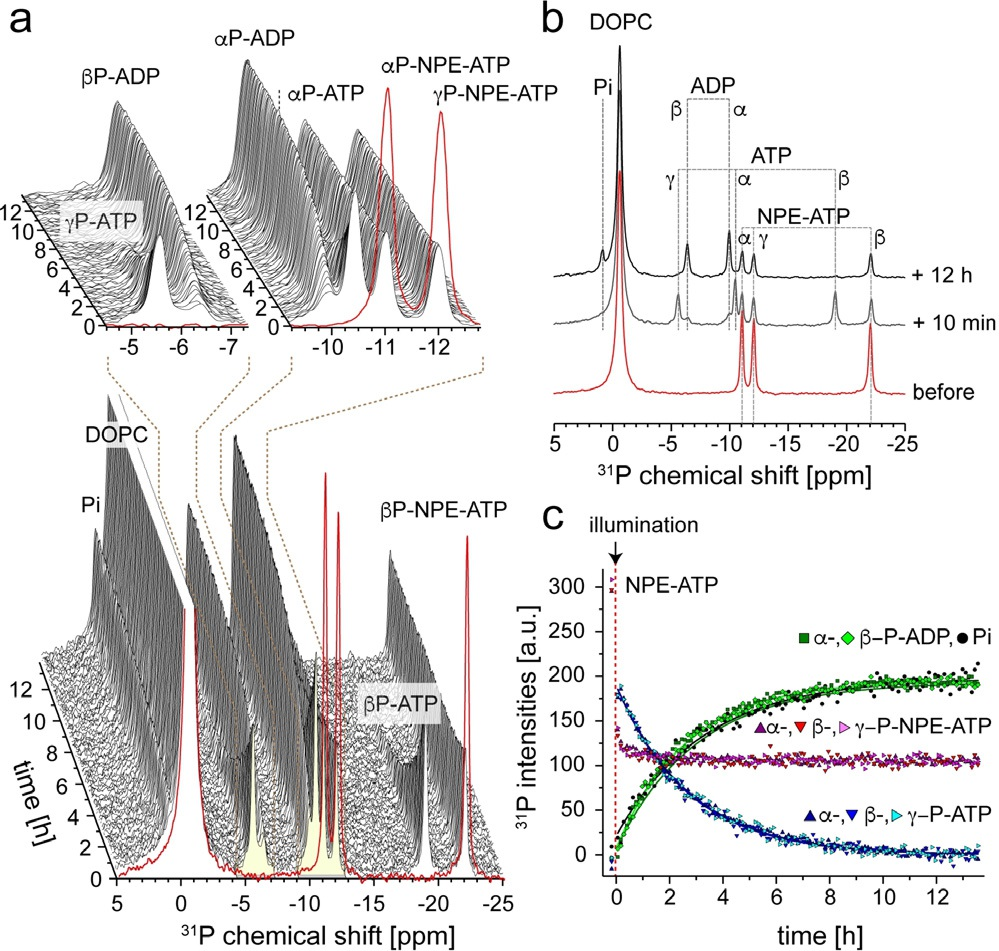
Uncaging of NPE‐ATP in the presence of DgkA containing DOPC proteoliposomes detected by ^31^P real‐time MAS NMR. a) NPE‐ATP gets uncaged to 65 % by 5 min illumination (see Figure S2). The released ATP is then turned over by DgkA into ADP and Pi. b) Comparison of ^31^P spectra before uncaging, directly after uncaging and at the end of the in (a) shown real‐time NMR experiment. c) Time traces of the in (a) performed real‐time experiment following DgkA's ATPase activity upon uncaging. The sample contained 300 nmol NPE‐ATP illumination in presence of 0.3 mg DgkA reconstituted into DOPC liposomes (molar lipid:protein ratio 120:1, 50 mm HEPES, pH 7.5, 30 mm NaCl and 2:1 molar ratio MgCl_2_:ATP). The sample volume was 15 μL. Spectra were recorded at 30 °C at a MAS rate of 10 kHz.

The data in Figure 2 demonstrate the feasibility of triggering an enzymatic reaction by light‐induced uncaging of NPE‐ATP in the presence of DgkA proteoliposomes. In order to generalize this approach, it would be desirable to bring also a long‐chain diacyclglycerol lipid substrate such as dioleoylglycerol (DOG) under light control. It is highly hydrophobic and cannot be added by simple mixing but would have to be incorporated already at the stage of liposome formation.^11^ DOG was therefore protected with an NPE group at the hydroxyl moiety, which prevents phosphorylation by DgkA without uncaging.^3^ The NPE group was connected via an oxycarbonyl linker, initially used for caging nucleoside 5′‐hydroxyls, in order to enhance the uncaging efficiency as hydroxyls are poor leaving groups compared to phosphates (see SI for further details on the synthesis).^12^

Unlike NPE‐ATP, NPE‐DOG has no ^31^P spectroscopic marker as direct NMR‐readout for successful uncaging via solid‐state NMR. However, successful uncaging can be shown indirectly: In the presence of lipid substrate, the basal ATPase activity of DgkA gets stimulated and turns into a phosphoryl transfer reaction.^5^ As a result, an increase in ATP consumption but a decrease in Pi production is observed as the γ‐phosphoryl group is transferred to DOG. This is indeed the case upon illumination of a sample containing DgkA within DOPC and NPE‐DOG bilayers (Figure S4): Upon addition of Mg.ATP, ATPase activity is observed by the build‐up of Pi. Irradiation with UV light uncages NPE‐DOG, which leads to an increase in ATP turnover but not Pi.

The subsequent phosphorylation of DOG is difficult to detect as its ^31^P signal is partially covered by the DOPC resonance. We have therefore repeated the experiment but replaced Mg.ATP by Mg.ATPγS as nucleotide substrate. Here, the thiophosphoryl group of ATPγS is transferred to uncaged DOG and the resulting lipid product thiophosphatidic acid (ThioPA) is significantly downfield shifted from the main lipid resonance. Indeed, after illumination and uncaging of NPE‐DOG, an increasing signal at 44 ppm is detected confirming successful uncaging of NPE‐DOG and subsequent phosphorylation of the released DOG within the membrane (Figure 3 and Figure S5). The small ThioPA signal observable before illumination causes a baseline offset and can be attributed to slightly incomplete caging of DOG, which has been thiophoshorylated upon addition of ATPγS during the dead time of the experiment. Additional purification steps might further decrease the fraction of lipid educt if required.^3^

**Figure 3:**
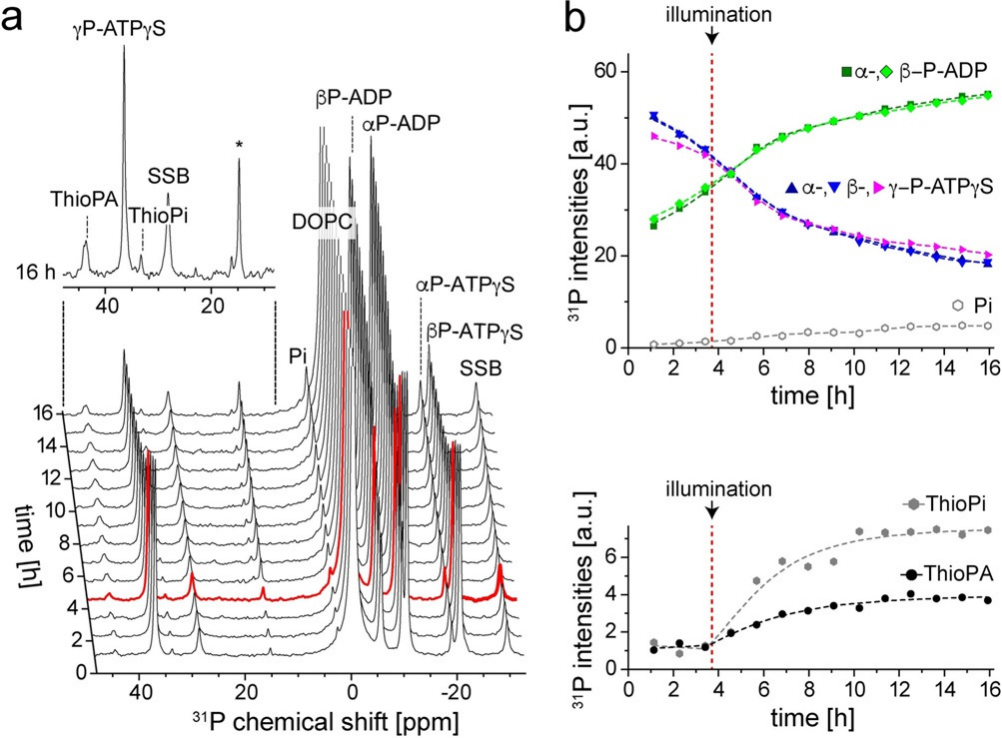
Light‐induced uncaging of NPE‐DOG in the presence of DgkA yields thiophosphatidic acid upon addition of Mg.ATPγS. a) Stacked plot of the ^31^P real‐time MAS NMR experiments on DgkA in DOPC liposomes containing NPE‐DOG and ATPγS. Basal ATPase activity is observed. Upon illumination for 5 min performed during acquisition of the spectrum marked in red, NPE‐DOG gets uncaged and an increasing signal of ThioPA is observed at 44 ppm. The asterisk denotes a thiophosphate (ThioPi) side‐product (see Figure S5b). b) Time traces of the ^31^P real‐time NMR experiment depict basal ATPase activity before uncaging of NPE‐DOG and kinase activity in conjunction with enhanced ATPase activity after uncaging as seen by formation of ThioPA and enhanced built‐up of ThioPi (mainly observed in form of its side‐product (*)). The sample contained 75 nmol ATPγS and 13.3 μg DgkA reconstituted into DOPC liposomes with 20 mol % NPE‐DOG (L:P 2000:1, 50 mm HEPES, pH 7,5, 30 mm NaCl and 2:1 molar ratio MgCl_2_:ATP). The sample volume was 15 μL. Spectra were recorded at 30 °C at a MAS rate of 10 kHz.

Hydrolysis of ATPγS by DgkA is known to be an order of magnitude faster compared to ATP.^5^ Therefore, stimulated hydrolysis of ATPγS upon uncaging could also be seen in these experiments as build‐up of thiophosphate increased after illumination. However, the amount of lipid product formed after illumination reveals that only a small fraction of DOG (4 % with respect to the amount of incorporated NPE‐DOG, calculated by comparison with the DOPC integral) was thiophosphorylated. One reason is probably limited accessibility of the DgkA binding sites for uncaged DOG. An additional factor could be insufficient uncaging of NPE‐DOG. It can be assumed that uncaging of NPE‐DOG is less efficient compared to NPE‐ATP as primary alcohols caged with carbonate derivatives of highly efficient Coumarin based photocages exhibit a poor photolysis efficiency compared to γP caged ATP derivatives.^13^

Despite efficient illumination of the small active volume of the MAS rotors used by high‐performance LEDs, uncaging is relatively slow and incomplete. One reason is probably the relatively high concentration (>20 mm with respect to total sample volume) of the caged compounds within the liposome sample in the MAS rotor and subsequent competing light absorption by the leaving group. It has been demonstrated under solution‐state NMR conditions, that submillimolar concentrations of caged compounds can be released within seconds by laser illumination setups capable of delivering several watts of radiant flux.2a, 14 A higher radiant flux could therefore also for MAS NMR experiments be beneficial to achieve higher uncaging efficiencies at reasonable concentrations and illumination times.

The performed experiments demonstrate that biochemical reactions studied by solid‐state NMR can be brought under light control using caged compounds. The main advantage of light triggered reactions as demonstrated by uncaging a lipid substrate will thereby lie on initiation of reactions that cannot be started by mixing or when components have to be prevented from reacting during sample preparation. This proof‐of‐concept illustrates that combining MAS‐NMR with uncaging strategies and illumination methods offers a new possibility for controlling biochemical reactions in situ.

## Experimental Section

### General

The *E. coli dgkA* wild‐type gene carrying a N‐terminal hexa‐His tag sequence was cloned from pSD005 into pET‐19b (Cat. 69 677‐3, Novagen) by changing the HindIII recognition sequence to NdeI and inserting the gene between the NcoI and NdeI recognition sequence.

Production and purification of DgkA were performed as described previously^15^ with minor changes mentioned below (for SDS‐PAGE see Figure S6). For solubilization and purification Empigen BB was replaced by OG in the same weight amounts. The HEPES concentration in all buffers was 50 mm and LiCl was replaced by 30 mm NaCl. The protein was eluted from the Ni‐NTA resin with 0.4 m imidazole, 0.1 % (w/v) DDM, 50 mm HEPES pH 7.5, 30 mm NaCl and 1 mm BHT. The yield for DgkA was typically 50 mg per liter of culture. For reconstitution into liposomes, DOPC and NPE‐DOG were dissolved in chloroform/methanol 3:1 (v/v), dried and dissolved in buffer (50 mm HEPES pH 7.5 and 30 mm NaCl). Liposomes were de‐stabilized with 3 mm DDM before performing freeze‐thaw cycles. The NPE‐DOG containing liposomes (100 mm HEPES pH 7.5 and 30 mm NaCl) were first sonicated for 10 min in an ultrasonic bath before addition of DDM and freeze‐thaw cycling. Purified DgkA was reconstituted into the liposomes at the desired lipid to protein molar ratio under slow stirring at room temperature. Detergent removal and imidazole removal was performed as described^7^ before. Proteoliposome samples were pelleted and resuspended at the desired concentration by vortexing before being transferred into a 3.2 mm sapphire MAS rotor.

Solid‐state NMR experiments were performed on a Bruker Avance III 850 WB spectrometer operating at 850.31 MHz ^1^H frequency using 10 kHz MAS rate and 30 °C. The sample temperature was referenced via KBr T_1_ relaxation measurements at the same spinning speed. To limit sample heating no decoupling was used. ^31^P solid‐state NMR spectra are referenced to H_3_PO_4_ using a chemical shift of 58.62 ppm for triethylphosphine as external reference. For ^31^P real‐time NMR measurements pH of ATP and ATPγS solutions was adjusted to 7.5. NPE‐ATP was dissolved in 100 mm HEPES pH7.5. MgCl_2_ was added to the nucleotide solutions in two‐fold molar excess as lower ratios lead to severe ^31^P line broadening of ATP and ADP signals. Nucleotide was added to the rotor containing the desired proteoliposome amount directly before measurements. In‐situ illumination was achieved using a modified 3.2 mm DVT MAS NMR probe containing a custom‐built 2 mm diameter LUV 70 μm fiber bundle with macor ferrules (Leoni Fibertech). A UV LED with 500 mW output power connected to a computer‐controlled LED driver (LCS‐0365‐11‐22, SLC‐AV02‐US, Mightex Systems) in combination with a lightguide adapter (LCS‐LGA22‐0515, Mightex Systems) was used for illumination at a peak wavelength of 365 nm (see Figure S1). To initiate photocleavage and induce the reaction, samples were typically illuminated for 5 min. The output power at the end of the fiber guide was determined with a laser thermal power sensor (P/N 1Z02146, Ophir) connected to a Nova Display power meter (1Z01500, Ophir). Time traces were generated by integration of the respective peak area and scaled to the amount of added nucleotide.

## Conflict of interest

The authors declare no conflict of interest.

## Supporting information

As a service to our authors and readers, this journal provides supporting information supplied by the authors. Such materials are peer reviewed and may be re‐organized for online delivery, but are not copy‐edited or typeset. Technical support issues arising from supporting information (other than missing files) should be addressed to the authors.

Supplementary
