# Supplementary material for: Light‐Induced Uncaging for Time‐Resolved Observations of Biochemical Reactions by MAS NMR Spectroscopy
Source: Chemistry. 2020 May 13;26(30):6789–92. doi: 10.1002/chem.202000770 (PMC7317521; doi:10.1002/chem.202000770)
Supplement: Supplementary file 1 — Supplementary [file CHEM-26-6789-s001.pdf]

Chemistry–A European Journal

Supporting Information

Light-Induced Uncaging for Time-Resolved Observations of Biochemical Reactions by MAS NMR Spectroscopy

Julian de Mos,[a] Andreas Jakob,[b] Johanna Becker-Baldus,[a] Alexander Heckel,[b] and Clemens Glaubitz*[a]

Supporting Information

Light-induced uncaging for time-resolved observations of biochemical reactions by MAS NMR spectroscopy

Julian de Mos,[a] Andreas Jakob,[b] Johanna Becker-Baldus,[a]

Alexander Heckel,[b] and Clemens Glaubitz*[a]

[a] Institute for Biophysical Chemistry and Centre for Biomolecular Magnetic Resonance
[b] Institute for Organic Chemistry and Chemical Biology
Goethe University Frankfurt Max-von-Laue-Str. 9, 60438 Frankfurt am Main (Germany)
(*) glaubitz@em.uni-frankfurt.de

Reagents

Triethylphosphine sulfide (Cat. 30392.04, Lot D06K25) was purchased from Alfa Aesar (Haverhill MA, USA). DNase I (Cat. A3778, Lot 3O009479), 1,4-dithiothreitol (DTT, Cat. A1101, Lot 1M007995), D(+)-glucose (Cat. A1422, Lot 3L009476), glycine (Cat. A3707), 4-(2-Hydroxyethyl)piperazine-1-ethanesulfonic acid sodium salt (HEPES, Cat. A1070, Lot 1E009339), imidazole (Cat A1073, Lot 4H015478), isopropyl β-D-1-thiogalactopyranoside (IPTG, Cat. A1008, Lot 6501055), KH_2PO_4 (Cat. A1043, Lot 6U014139), lysozyme (Cat. A3711, Lot 8N010154), $MgCl_2 \cdot 6H_2O$ (Cat. A1036, Lot 7T000254), NaCl (Cat. A1149, Lot 4M014937), *n*-octyl-β-D-glucopyranoside (OG, Cat. A1010, Lot 6P012262) were purchased from AppliChem (Darmstadt, Germany). 1-2-dioleoyl-*sn*-glycerol (DOG, Cat. 800811, Lot 800811-01-058), 1,2-dioleoyl-*sn*-glycero-3-phosphocholine (DOPC, Cat. 850375P, Lot 181PC-264) were purchased from Avanti Polar Lipids (Alabaster AL, USA). Bio-Beads SM-2 (Cat. 152-3920, Lot 64162628) were purchased from Bio-Rad (Hercules CA, USA). Ampicillin sodium salt (Cat. K029.2, Lot 164213247), LB broth (Cat. X968.4, Lot 416250436) were purchased from Carl Roth (Karlsruhe, Germany). *n*-Dodecyl β-D-maltoside (DDM, Cat. D97002-C, Lot MR2807/4) was purchased from Glycon Biochemicals (Luckenwalde, Germany). Ni-NTA (Cat. 1018240, Lot 148024809) was purchased from Qiagen (Hilden, Germany). Complete protease inhibitor cocktail tablets (Ref 11836145001, Lot 11767900), was purchased from Roche (Basel, Switzerland). Adenosine 5′-triphosphate disodium salt (ATP, Cat. A26209, Lot MKBW6572V), adenosine 5′-[γ-thio]triphosphate tetralithium salt (ATPγS, Cat. A1388), tris(2-carboxyethyl)phosphine hydrochloride (TCEP, Cat C4706, Lot 095K1188) were purchased from Sigma-Aldrich (St Louis MO, USA). Adenosine 5′-triphosphate, P^3-(1-(2-nitrophenyl)ethyl) ester disodium salt (NPE-ATP, Cat. A1048, Lot 1644953, 1711735, 1780843, 1798352) was purchased from Thermo Fisher Scientific (Waltham MA, USA). Centrum vitamin pills (product no. 14006-01, Lot AVB024) were purchased from Pfizer Consumer Healthcare (Berlin, Germany).

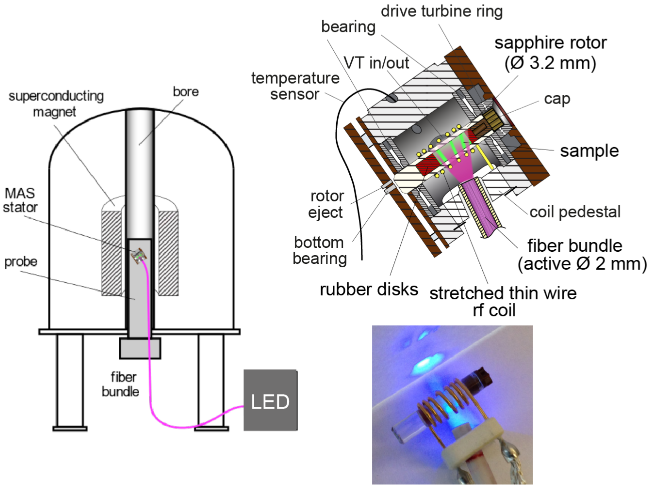

Figure S1: Experimental setup for *in situ* sample illumination under MAS NMR conditions. A Bruker 3.2 mm DVT MAS probe was equipped with a custom-built 2 mm diameter LUV 70 µm fiber bundle with macor ferrules (Leoni Fibertech). The NMR coil was slightly stretched in order to achieve better sample illumination. A UV LED with 500 mW output power and a peak wavelength of 365 nm (Mightex Systems) was used as light source. Samples were transferred into sapphire MAS rotors with rubber discs to restrict the volume to the center. The general experimental design is similar to the illumination setup described before for photo-CIDNP or DNP-enhanced MAS-NMR.[1]

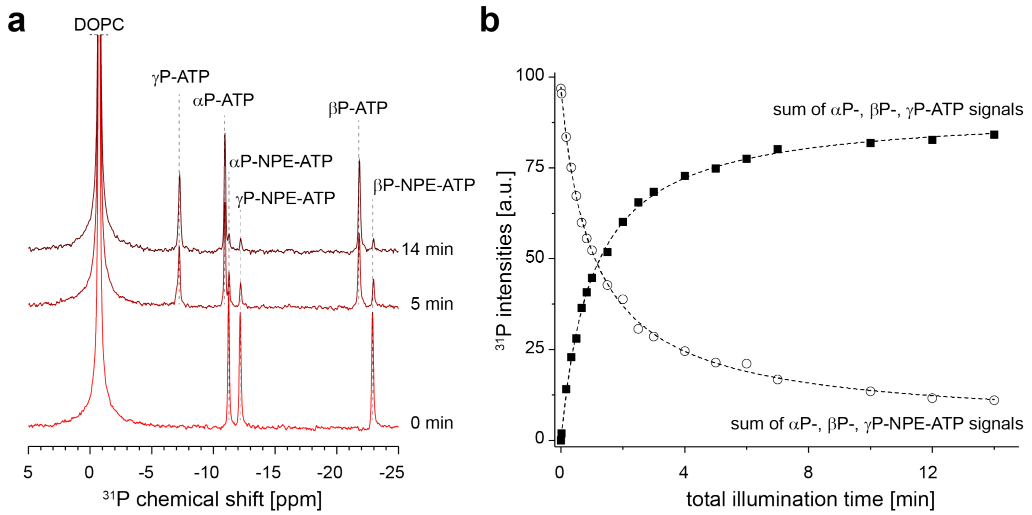

Figure S2: Demonstration of successful uncaging of NPE-ATP in the presence of liposomes under MAS-NMR conditions. **a)** ^{31}P-spectra of NPE-ATP recorded after 0 min, 5 min and 14 min illumination. **b)** ^{31}P progress curve for light-induced uncaging of NPE-ATP as a function of total illumination time. Data points were derived from 1D ^{31}P spectra as shown in (a) by summing up the integral ^{31}P peak intensities for α-, β- and γ-P for both compounds. After an initially fast release, uncaging slows down with time, after 14 min 23% NPE-ATP remain. The reason for this observation is increasing absorption caused by the leaving group at the same wavelength needed for triggering the uncaging reaction (365 nm).[2] Therefore, a non-linear decrease of the uncaging efficiency with time occurs. The sample contained 400 nmol NPE-ATP added to 3 mg DOPC in 50 mM HEPES pH 7.5 and 30 mM NaCl. The sample volume was 15 µl. Spectra were recorded at 30 °C at a MAS rate of 10 kHz.

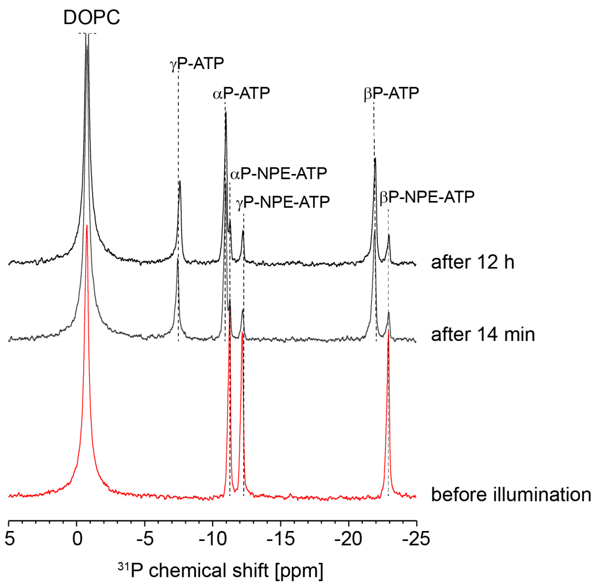

Figure S3. Uncaging of NPE-ATP in the presence of DgkA containing DOPC proteoliposomes but without Mg^{2+} followed by ^{31}P real-time MAS NMR. NPE-ATP gets uncaged to 65% by 5 min illumination but no ATP hydrolysis is observed. As DgkA has an absolute cofactor requirement for a divalent cation[3] and Mg^{2+} does not catalyze ATP hydrolysis in solution[4], the lack of ATP hydrolysis in absence of $MgCl_2$ confirms that DgkA catalyzes the in Figure 2 observed ATP hydrolysis in absence of a lipid substrate. The sample contained 0.3 mg DgkA in DOPC liposomes at a molar ratio to 1:120 and 300 nmol NPE-ATP (50 mM HEPES, pH 7.5, 30 mM NaCl, $MgCl_2$: ATP = 2 :1 mole/mole). The sample volume was 15 µl. Spectra were recorded at 30 °C at a MAS rate of 10 kHz.

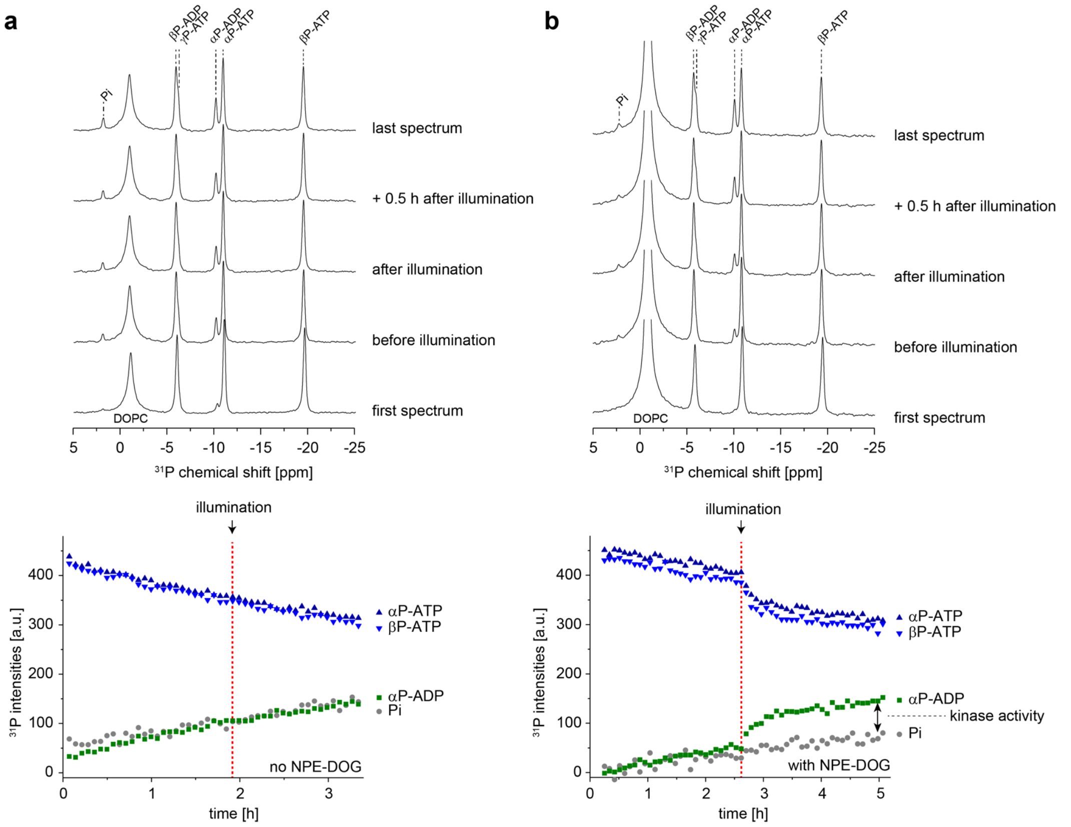

Figure S4. Indirect detection of NPE-DOG uncaging. **a)** Selected spectra of ^{31}P real-time MAS NMR experiments and corresponding time traces following DgkA's activity in liposomes upon addition of Mg.ATP. Illumination for 5 min during acquisition does not enhance the enzymatic activity of DgkA reconstituted in DOPC liposomes without NPE-DOG. **b)** In contrast, illumination for 5 min during acquisition triggers an enhanced ATP turnover in a sample containing DgkA reconstituted into DOPC liposomes with 20 mol% NPE-DOG. The enhanced ATP turnover does not result in an increased Pi built-up upon illumination because the cleaved ATP γ-phosphoryl group is transferred to DOG.
The sample in a) contained 450 nmol ATP, 54 µg DgkA reconstituted into DOPC liposomes (L:P 480:1, 50mM HEPES, pH7,5, 30mM NaCl, 900 nmol $MgCl_2$). The sample in b) contained 450 nmol ATP, 40 µg DgkA reconstituted into DOPC liposomes containing 20 mol% NPE-DOG (L:P 2000:1, 50mM HEPES, pH7,5, 30mM NaCl, 2:1 molar ratio $MgCl_2$: ATP). The sample volume was 15 µl. Spectra were recorded at 30 °C at a MAS rate of 10 kHz.

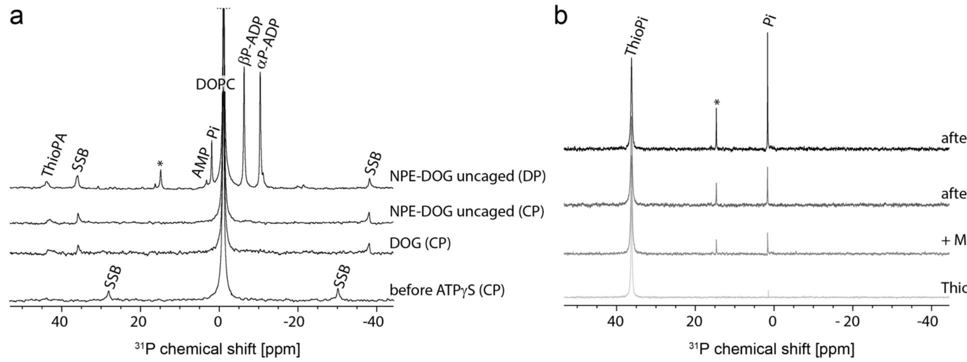

Figure S5. Formation of thiophosphatidic acid (ThioPA) by DgkA with ATPγS as nucleotide substrate. **a)** Comparison between ^{31}P direct polarized (DP) and cross-polarized (CP) spectra of DOG and uncaged NPE-DOG recorded after the reactions has completed. In both cases, a resonance at 44 ppm is observed demonstrating that ThioPA has formed. The spectrum before addition of ATPγS to the sample containing NPE-DOG has been recorded with 10 kHz MAS at 20.0 Tesla, 344 MHz ^{31}P. The other spectra were recorded at 14.1 Tesla, 243 MHz ^{31}P with 9 kHz MAS to avoid overlap with spinning side bands. **b)** The signal denoted with an asterisk in (a) and Figure 3a arises from thiophosphate in presence of $MgCl_2$ as can be seen by ^{31}P liquid-state NMR spectra of thiophosphate recorded with two-fold molar excess of $MgCl_2$ at pH 7.5 at different times (7.05 Tesla, 121 MHz ^{31}P).

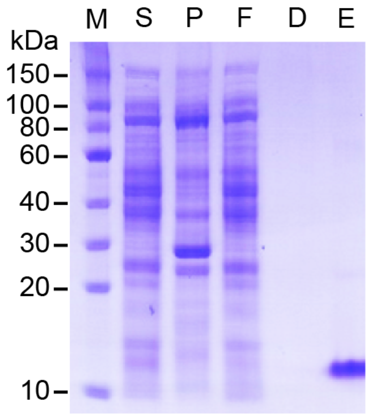

Figure S6. SDS-PAGE (Coomassie stain) of DgkA: (M) protein marker 10-150 kDa, (S) supernatant, (P) pellet, (F) Ni-NTA column flow-through, (D) 7.5 µl of 100 ml OG to DDM detergent exchange wash, (E) eluate containing 2 µg DgkA.

Synthesis of 3-((1-(2-nitrophenyl)ethoxy)carbonyl)-sn-1,2-dioleoylglycerol (NPE-DOG)

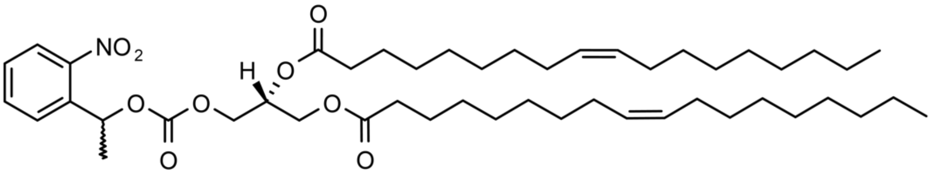

$C_{48}H_{79}NO_9$

M = 814.15 g/mol

Figure S7. DOG (1-2-dioleoyl-*sn*-glycerol) was bought from Avanti Polar Lipids as oil. DOG (0.1 g, 0.16 mmol, 1.0 eq) with DIPEA (0.62 g, 0.48 mmol, 3.0 eq) was stirred into a mixture of DCM/Pyrimidin (1 ml) at 0 °C. 1-(2-nitrophenyl) chloroformate (0.057 g, 0.25 mmol, 0.5 eq) synthesized as described[5] was used without further purification. (0.057 g, 0.25 mmol, 0.5 eq) were dissolved in DCM (1 ml) and added to the reaction mixture. The solution was stirred for 3 days at room temperature. The product was extracted with EtOAc, saturated aqueous NaCl solution and H_2O. Combined organic phases were dried over Na_2SO_4 and solvent removed *in vacuo*. Purification was performed via column chromatography with CH/EtOAc (5:1). A yield of 0.1 g (50 %) was achieved. The product was analyzed by MALDI-HRMS. The m/z calculated for $C_{48}H_{79}NO_9$ $[M+Na]^+$ is 836.56470 Da and a value of 836.5649 Da (Δm 0.00028 Da, error 0.33 ppm) was obtained. ^1H and ^{13}C spectra are shown In Figures S8 and S9.

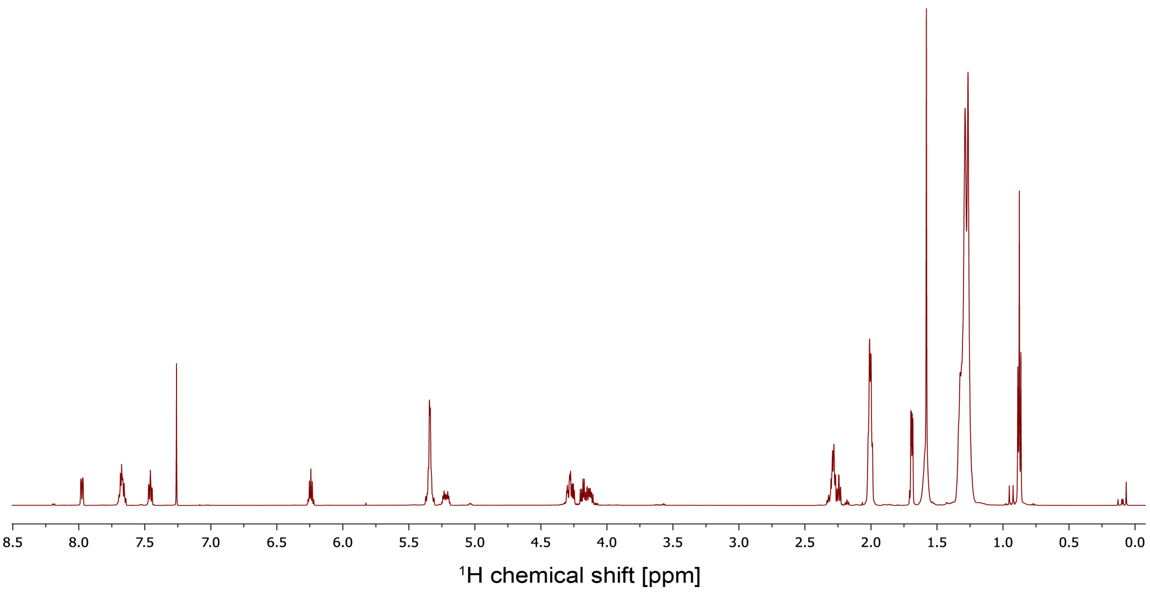

Figure S8. ^{1}H-NMR spectra of NPE-DOG in $CDCl_3$ (600 MHz): δ = 8.00 – 7.96 (m, 1H), 7.71 – 7.64 (m, 2H), 7.48 – 7.44 (m, 1H), 6.28 – 6.21 (m, 1H), 5.39 – 5.30 (m, 4H), 5.26 – 5.18 (m, 1H), 4.35 – 4.24 (m, 2H), 4.21 – 4.10 (m, 2H), 2.34 – 2.22 (m, 4H), 2.01 (dd, *J* = 12.8, 6.4 Hz, 8H), 1.69 (dd, *J* = 6.5 Hz, 3.1 Hz, 3H), 1.64 – 1.56 (m, 4H), 1.36 – 1.22 (m, 40H), 0.88 (t, *J* = 7.0 Hz, 6H) ppm.

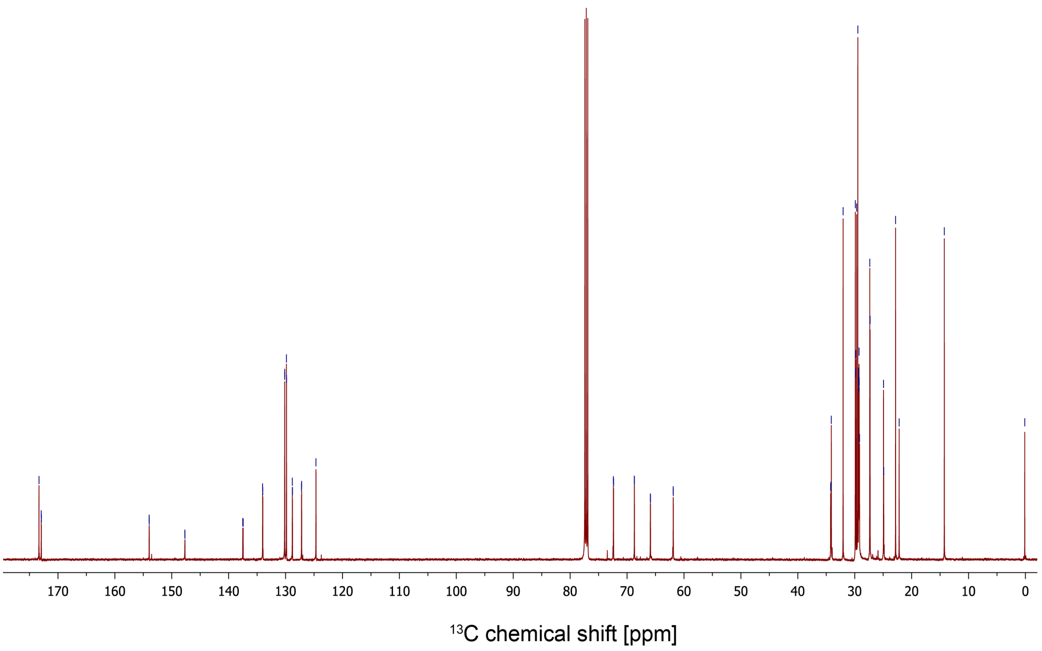

Figure S9. ^{13}C-NMR spectra of NPE-DOG in $CDCl_3$ (126 MHz): δ = 173.20, 172.79, 153.83, 147.56, 137.36, 133.87, 130.03, 129.71 128.67, 127.06, 124.53, 72.26, 68.58, 65.77, 61.75, 33.98, 31.91, 29.77, 29.73, 29.71, 29.53, 29.33 29.19,29.17, 29.13, 29.11, 29.08, 29.03, 27.23, 27.18, 24.81, 24.78, 22.69, 22.07, 14.13, 0.00 ppm.

References

[1] a) J. Becker-Baldus, C. Bamann, K. Saxena, H. Gustmann, L. J. Brown, R. C. Brown, C. Reiter, E. Bamberg, J. Wachtveitl, H. Schwalbe, C. Glaubitz, *Proc. Natl..Acad..Sci. U. S. A.* **2015**, *112*, 9896-9901; b) J. Matysik, Alia, J. G. Hollander, T. Egorova-Zachernyuk, P. Gast, H. J. de Groot, *Indian J. Biochem. Biophys.* **2000**, *37*, 418-423.

[2] a) A. Barth, J. E. T. Corrie, M. J. Gradwell, Y. Maeda, W. Mantele, T. Meier, D. R. Trentham, *J. Am. Chem. Soc.* **1997**, *119*, 4149-4159; b) C. H. Park, R. S. Givens, *J. Am. Chem. Soc.* **1997**, *119*, 2453-2463.

[3] E. G. Schneider, E. P. Kennedy, *Biochim. Biophys. Acta* **1976**, *441*, 201-212.

[4] a) D. Herschlag, W. P. Jencks, *J. Am. Chem. Soc.* **1987**, *109*, 4665-4674; b) N. H. Williams, *J. Am. Chem. Soc.* **2000**, *122*, 12023-12024.

[5] S. Tang, Z. Wan, Y. Gao, J.-S. Zheng, J. Wang, Y.-Y. Si, X. Chen, H. Qi, L. Liu, W. Liu, *Chemical science* **2016**, *7*, 1891-1895.
